# Reciprocal relationships between personality disorders and eating disorders in a prospective 17‐year follow‐up study

**DOI:** 10.1002/eat.23823

**Published:** 2022-10-10

**Authors:** Hanna Punsvik Eielsen, KariAnne Vrabel, Asle Hoffart, Øyvind Rø, Jan H. Rosenvinge

**Affiliations:** ^1^ Research Institute, Modum Bad Psychiatric Centre Vikersund Norway; ^2^ Department of Psychology University of Oslo Oslo Norway; ^3^ Division of Mental Health and Addiction, Regional Department for Eating Disorders Oslo University Hospital Oslo Norway; ^4^ Division of Mental Health and Addiction, Institute of Clinical Medicine University of Oslo Oslo Norway; ^5^ Department of Psychology, Faculty of Health Sciences UiT ‐ The Arctic University of Norway Tromsø Norway

**Keywords:** eating disorders, follow‐up, multilevel modeling, outcome, personality disorders

## Abstract

**Objective:**

This study aimed to report the presence of categorical and dimensional personality disorders (PD) in adults with longstanding eating disorders (ED) over a period of 17 years and to investigate whether changes in PD predict changes in ED symptoms or vice versa.

**Methods:**

In total, 62 of the 80 living patients (78% response rate) with anorexia nervosa (*n* = 23), bulimia nervosa (*n* = 25), or other specified feeding or ED (*n* = 14) at baseline were evaluated during hospital treatment and at 1‐year, 2‐year, 5‐year, and 17‐year follow‐up. PD were assessed using the Structured Clinical Interview for DSM‐IV Axis II disorders, and the eating disorder examination (EDE) interview was used to assess ED. Data were analyzed using multilevel modeling.

**Results:**

From baseline to the 17‐year follow‐up, the number of patients with any PD decreased significantly from 74.2% to 24.2%, and the total number of PD diagnoses declined from 80 to 22. Mean EDE score was significantly reduced from 4.2 (SD: 1.1) to 2.0 (SD: 1.6). There was a positive association between ED and PD where the initial level of either disorder was followed by similar levels of the other disorder throughout the entire follow‐up period. High baseline levels of borderline PD predicted less decrease in ED symptoms. No significant within‐person effects were found.

**Conclusions:**

Both ED and PD significantly declined over time. As the severity of either disorder seems to be associated with the other, thorough assessment and treatment that incorporates both the ED psychopathology and the personality disturbances are advisable.

**Public Significance Statement:**

While personality disorders were highly prevalent in the sample of patients with longstanding eating disorders, both disorders were significantly reduced at the 17‐year follow‐up. The disorders are related in the sense that an initial high level of either disorder is associated with a high level of the other over time. A thorough assessment and attention to both illnesses are advisable in therapy.

**Clinical Trial Identifier:**

NCT03968705.

## 1 INTRODUCTION

Several meta‐analyses (Friborg et al., 2014; Martinussen et al., 2017; Rosenvinge et al., 2000) have shown significantly higher proportions of personality disorders (PD) in patients with eating disorders (ED) than in healthy controls. Comorbid PD in patients with ED are associated with increased levels of general psychopathology despite high treatment utilization (Cassin & von Ranson, 2005; Keel et al., 2002; Voderholzer et al., 2021; Zeeck et al., 2007), making this a matter of clinical importance. A recent systematic review (Simpson et al., 2021) revealed mostly unfavorable treatment outcomes for ED, possibly because ED symptoms tend to become more severe and intractable when occurring alongside a PD. The comorbidity of PD and ED may initiate self‐reinforcing feedback, exacerbating the role of ED symptoms as dysfunctional affect regulating and coping strategies (Helverskov et al., 2010; Sansone & Sansone, 2010; Simpson et al., 2021). Similarly, PD traits like impulsivity, perfectionism, rigidity, and interpersonal problems may delay or compromise ED recovery by increasing the risk of reduced therapeutic alliance and premature drop‐out from treatment (Chen et al., 2011; Hessler et al., 2019; Pham‐Scottez et al., 2012).

Considering the long duration of both PD and ED, time is a critical factor to take into account when examining the interplay between the two. To identify patterns of stability and change in personality pathology in samples of patients with ED, there is a need for prospective longitudinal studies with repeated assessments using structured clinical interviews at different stages in the ED recovery process (Grilo et al., 2003; Magallón‐Neri et al., 2014; Reas et al., 2013). However, prospective studies of this kind are both scarce and heterogeneous in terms of assessment methods and number of measuring points. Furthermore, they are largely limited to a short or intermediate observation time. Among studies with an intermediate follow‐up (3–5 years) after ED treatment, PD, particularly borderline and avoidant PD, have been related to higher psychological distress, though unrelated to ED remission (Rowe et al., 2008, Rowe et al., 2010, 2011; Wonderlich et al., 1994). Grilo et al. (2007) utilized a dimensional assessment of PD in their 5‐year prospective study; they found no relation to ED remission, with the exception of avoidant PD. In that case, each increase in number of fulfilled criteria decreased the chances of ED recovery. This illustrates how a dimensional approach facilitates more nuance, while cutoffs and thresholds might lead to loss of information. A combination of dimensional and categorical measures is therefore needed to increase clinical validity (Vrabel, Hoffart, et al., 2010). Few studies have examined the PD–ED relationship beyond the intermediate follow‐up period. A 10‐year follow‐up study that investigated ED and borderline PD comorbidity (Zanarini et al., 2010) through structured diagnostic interviews at five assessment points found high rates of remission from ED, but both relapse and migration between ED diagnoses were common. Studies with a similar or longer timeframe have shown that poor prognosis is associated with an avoidant‐insecure personality type (Thompson‐Brenner et al., 2008), a history of personality disturbances (Ratnasuriya et al., 1991), premorbid PD (Rigaud et al., 2011), premorbid obsessive–compulsive PD (Wentz et al., 2009), and severity of social and psychological problems (Löwe et al., 2001). However, none of these studies utilized structured diagnostic interviews for repeated assessment of PD.

Furthermore, follow‐up studies of patients with ED have focused on between‐person differences (Rowe et al., 2010). This typically means that a patient's initial level of, for example, impulsivity relative to other patients' levels of impulsivity at that time is used to predict the subsequent outcome score, such as the patient's ED symptoms relative to the other patients' ED symptoms at the follow‐up assessment. However, a clinician is generally less interested in how a patient is doing relative to other patients than in whether the patient is doing better or worse compared with their own typical state. This level of inquiry relies on multiple assessments of the same constructs within the same patients over time. By utilizing all the available data, one can estimate an expected level of the construct of interest and calculate the patient's deviation from that estimate at any assessment point, thereby generating information about the degree to which the patient is different from their norm. By applying such analyses more of the complexity in the ED–PD comorbidity might be revealed. In addition to introducing innovative analyses, this study also added a fifth assessment, using structured diagnostic interviews 17 years after admission, thereby extending the prospective study of patients with longstanding ED (Rø et al., 2003, 2004, 2005; Vrabel et al., 2008). Previously, structural equation modeling analyses demonstrated that improvements in ED symptoms preceded the reduction of personality psychopathology (Rø, Martinsen, Hoffart, Sexton et al., 2005). This may indicate that PD symptoms were at least partially caused by the ED symptoms; however, the decline in both ED and PD symptoms continued for the group altogether at both the 2‐year (Rø et al., 2005) and the 5‐year follow‐up (Vrabel, Hoffart, et al., 2010). Notably, a poor outcome was found in a subsample of patients with a combination of avoidant PD and a history of childhood sexual abuse (Vrabel, Rø, et al., 2010). Extending the observation period from the previous assessment by more than a decade might increase our understanding of outcome predictors for these long‐lasting disorders. As the search for predictors of ED outcome has generated mixed findings, it is crucial to control for several variables to avoid conclusions based on confounding factors. Body mass index (BMI) is highly relevant, as malnutrition and underweight might be related to symptoms such as obsessive–compulsive behavior and rigid mindset (Friborg et al., 2014), and higher proportions of PD have been found in samples categorized as underweight compared with samples of patients with normal‐ or overweight (Martinussen et al., 2017). Treatment duration (Herzog et al., 1999), illness duration (Fichter et al., 2017), and age (Dobrescu et al., 2020; Martinussen et al., 2017) are all related in the sense that higher age tends to be associated with longer illness duration and possibly prolonged therapy, both of which are generally associated with poor outcome (Fichter et al., 2017).

This study aimed to (1) report the presence of categorical and dimensional PD in adults with a history of longstanding ED upon hospital treatment and at 1‐year, 2‐year, 5‐year, and 17‐year follow‐up, respectively; and (2) investigate whether changes in PD predict changes in ED symptoms, or vice versa, at both the within‐person and between‐person level.

## 2 METHODS

### 2.1 Sample

All patients ≥18 years of age with anorexia nervosa (AN), bulimia nervosa (BN), or other specified feeding and ED (OSFED) and no response to previous ED treatment, who were consecutively admitted to a specialized ED unit at Modum Bad Psychiatric Hospital in Norway from August 1998 to June 2001 were eligible for the study. The patients participated in a multicomponent 15‐week (BN/OSFED) or 23‐week (AN/OSFED) inpatient program based on cognitive behavioral therapy conducted in both group and individual sessions. Patients with a combination of severe somatic complications and a BMI < 14 were referred elsewhere for medical stabilization. Details of the treatment program are provided elsewhere (Rø et al., 2004; Rø et al., 2003), along with outcomes at 2 years (Rø et al., 2005), 5 years (Vrabel et al., 2008), and 17 years (Eielsen et al., 2021). Of the 92 patients admitted to the unit during this time period, 86 (one assigned male at birth, the remaining assigned female), chose to participate in the longitudinal study. All participants were Caucasian, more specifically Scandinavian, as identified by self‐report, and all were Norwegian‐speaking. According to data from the Norwegian Cause of Death Registry, six patients (5.6%) from the original sample were deceased. For time course and reasons of attrition (see Supplemental Appendix S1). At the 17‐year follow‐up, 62 patients agreed to participate. At admission, 23 patients (37.1%) had AN, 25 (40.3%) had BN, and 14 (22.6%) had OSFED, and the average illness duration was 12.9 years (SD: 7.4, range: 2–33). The mean BMI for the different ED subgroups were 16.5 (SD: 1.7) for AN, 22.0 (SD: 3.4) for BN, and 21.8 (SD: 5.0) for OSFED, with a mean total of 19.9 (SD: 4.2). The patients had received previous psychiatric treatment for a mean of 2.8 years (SD: 2.2, range: 0–10), of whom 35 (56.5%) in terms of inpatient treatment. In addition, 33 patients (53.2%) had a history of self‐mutilation and 25 (40.3%) had previously attempted suicide. The mean age at admission was 29.3 (SD: 7.2, range: 19–47). The majority of the patients were heterosexual (95.2%), 35 patients (56.5%) had a partner, and 15 patients (24.2%) had children. In total, 41 patients (66.1%) were either on sick leave or receiving other welfare benefits, whereas 21 (33.9%) were working or studying at the time of admission.

### 2.2 Assessments

#### 2.2.1 Personality disorders

The Structured Clinical Interview for DSM‐IV Axis II diagnoses (SCID‐II; First et al., 1997) was used to identify PD diagnoses and PD dimensions. The SCID‐II assessment at baseline was conducted near the end of the inpatient treatment, aiming to reduce the risk of false positive PD diagnoses due to emotional or cognitive consequences of starvation, or impulsivity related to binge eating episodes (Pollice et al., 1997). The presence of a given PD was determined by a varying number of items for each diagnosis. Each item was scored using a 3‐point scale (threshold = 3, subthreshold = 2, and absent = 1). Categorical diagnoses were obtained when the number of “3” scores reached the DSM‐IV diagnostic threshold. Dimensional indices were calculated by adding the 1–3 scores and dividing the sum by the number of items (Hoffart & Hedley, 1997). Both the categorical and dimensional PD, as measured by SCID‐II, have excellent interrater agreement (Lobbestael et al., 2011). Reliability varied from good to unacceptable for the five most frequently observed diagnoses across assessments: Avoidant (Cronbach's *α* = .83–.89), dependent (Cronbach's *α* = .48–.67), obsessive–compulsive (Cronbach's *α* = .48–.73), paranoid (Cronbach's *α* = .43–.75), and borderline PD (Cronbach's *α* = .65–.79). The low *α* values are likely related to the low incidence of the different PD. Therefore, the statistical analyses only included avoidant and borderline PD, which had nearly acceptable to good *α* values at all measuring points.

#### 2.2.2 Eating disorders

The eating disorder examination (EDE) interview (Fairburn, 2008) was used to assess ED psychopathology and generate ED diagnoses. The EDE‐17 is the most recent version of the interview and is adapted to the DSM‐5 (American Psychiatric Association [APA], 2013). It consists of four subscales: “restraint,” “shape concern,” “weight concern,” and “eating concern.” A mean value is calculated on a 0‐point to 6‐point scale, referred to as global EDE. The data material collected using the EDE‐12 interview at previous assessments were re‐evaluated according to the updated diagnostic criteria, to enable comparison across measuring points. The Norwegian translation has adequate internal consistency and interrater reliability (Reas et al., 2011). The Cronbach's *α* for the global EDE ranged from .82 to .96 across the four assessments using EDE‐12, while the EDE‐17 had an *α* value of .94.

#### 2.2.3 Control variables

Information regarding age, duration of treatment, and illness duration were collected in baseline interviews. The patients' weight and height were measured at admission and were used to calculate BMI.

### 2.3 Procedure

The diagnostic interviews were conducted during inpatient treatment and at the 1‐year, 2‐year, 5‐year, and 17‐year follow‐up, respectively. The mean time from admission to 1‐year follow‐up was 1.0 year (SD: 0.2), to 2‐year follow‐up was 2.0 years (SD: 0.3), to 5‐year follow‐up was 5.5 years (SD: 1.0), and to 17‐year follow‐up was 17.0 years (SD: 1.2). At each measuring point, informed consent was collected from all participants. All the 17‐year interviews were recorded, and 15 tapes containing the SCID‐II interviews were randomly selected for blind rating by an expert clinician, the second author. Interrater agreement for PD diagnoses was evaluated by means of intraclass correlation coefficients (ICC; Shrout & Fleiss, 1979), and was good for borderline (*r =* .77) and excellent for avoidant (*r* = .94). There was a perfect kappa (*k*) of 1.00 for the presence of any PD, except for avoidant PD, which had a *k* of 0.76. The baseline diagnostics were conducted by the patients' individual therapists at the end of 15–23 weeks of hospital treatment. Interviews at the three earliest measuring points were performed by a total of 11 experienced psychologists, resident doctors, and psychiatrists. The fourth author conducted 50% of the interviews at the 1‐year and 2‐year follow‐up. At the 5‐year follow‐up, all interviews were conducted by the second author, except for one which was carried out by the fourth author. At the 17‐year assessment, 97% of the interviews were conducted by the first author, two by the second author, and one by the fourth author. All three are psychologists or psychiatrists with an average of 15 years of experience in the field of ED, and all are trained in diagnostic interviewing using SCID‐II.

### 2.4 Statistical analyses

Data were analyzed using SPSS version 27.0. The McNemar's test was used for bivariate analysis, and the interrater reliability was calculated with ICC and *k* (Shrout & Fleiss, 1979). Given the exploratory nature of the study, a significance level of <.05, two‐tailed, was selected for all analyses.

To examine the relationship between the global EDE and the dimensional PD over time, multilevel mixed models (MLM) were used to enable analysis of the two levels of data; repeated assessments nested within the patients (Krull & MacKinnon, 1999). This method can adjust for the interdependence of the repeated measurements by adding individual‐specific random effects and by modeling the covariance structure of the residuals. Only the two PD with the highest alpha values across assessments were included in the MLM analyses. The missing completely at random test (Little, 1988) was nonsignificant for all outcome measures; EDE (*χ* ^2^ = 3637.47, *p* = 1.000), avoidant (*χ* ^2^ = 167.18, *p* = .205), and borderline PD (*χ* ^2^ = 173.52, *p* = .123). This indicates that the data were missing completely at random. Missing data were handled using full maximum likelihood estimation (FIML), which allows individuals to contribute with the proportion of data they have available in the estimation procedure. As a state‐of‐the‐art approach in scenarios with missing data (Schafer & Graham, 2002), FIML is superior to other analytic methods because it yields less biased results (O'Connell et al., 2017).

The models were built on the basis of a model including only a fixed intercept and no random effects, and the −2 log‐likelihood ratio test was used to examine the model fit. More complexity was gradually added, first a random intercept, then a random effect of time. Different covariance structures were tested to find the model with the best fit. Also, different versions of the time scale were tested. Numeric time gave the best fit (0, 1, 2, 3, and 4) compared with both real time (calculated by each individual's date of admission subtracted from their individual date of each assessment, e.g., 0, 12, 25, 86, 224) and real time average (0, 12, 24, 60, and 204). A few iteration problems emerged as the analyses increased in complexity when within‐person and between‐ person predictors were added. This was solved by simplifying the models to the best one that converged. The EDE model with borderline PD as a predictor included no random effects. First‐order autoregressive structure was the best fitting covariance structure of the residuals. When using avoidant PD as a predictor in the EDE model, random time was added, with variance components as a covariance structure.

When examining EDE as a predictor of PD, the model for avoidant PD included a random intercept with an unstructured covariance structure. The random intercept accounts for variability in participants' baseline scores. The model for borderline PD, with EDE as a predictor, included no random effects, and a heterogeneous first‐order autoregressive covariance structure gave the best fit for the residuals. Key model assumptions (e.g., normality of the residuals) were tested. The initial models included other fixed effects that were considered important to control for, all of which are well‐known potential predictors of outcome: BMI, treatment duration, illness duration, and age. Following the recommendation of Wang and Maxwell (2015) we did not control for time as the long‐term course was our main interest. The interaction with time, however, was tested for all variables, to explore potential changes in the ED–PD relationship over time.

Because there was a linear trend over time in the outcome variables, the disaggregation method of detrending was used to separate the within‐person and between‐person variance components of the predictors (Curran & Bauer, 2011). The between‐person component was estimated as the intercept (i.e., the expected value at the first assessment). The within‐person component was estimated as the deviation from the expected regression line for the individual patient at each assessment, that is, the residuals at each measuring point from the linear growth line. All the within‐person predictor scores (global EDE and the two PD indices) were lagged to establish a temporal sequence so that the variables could predict scores at the subsequent assessment.

### 2.5 Ethical considerations

This research complied with the Helsinki Declaration and was approved by the Regional Committees for Medical and Health Research Ethics (REC), Norway (REC‐number 2010/2548). The study was conducted in compliance with APA ethical standards and IRB standards for research involving human subjects. Written informed consent was obtained from all patients. The study's design is registered on clinicaltrials.gov (clinical trial number NCT03968705).

## 3 RESULTS

### 3.1 Presence of categorical and dimensional PD

**FIGURE 1:**
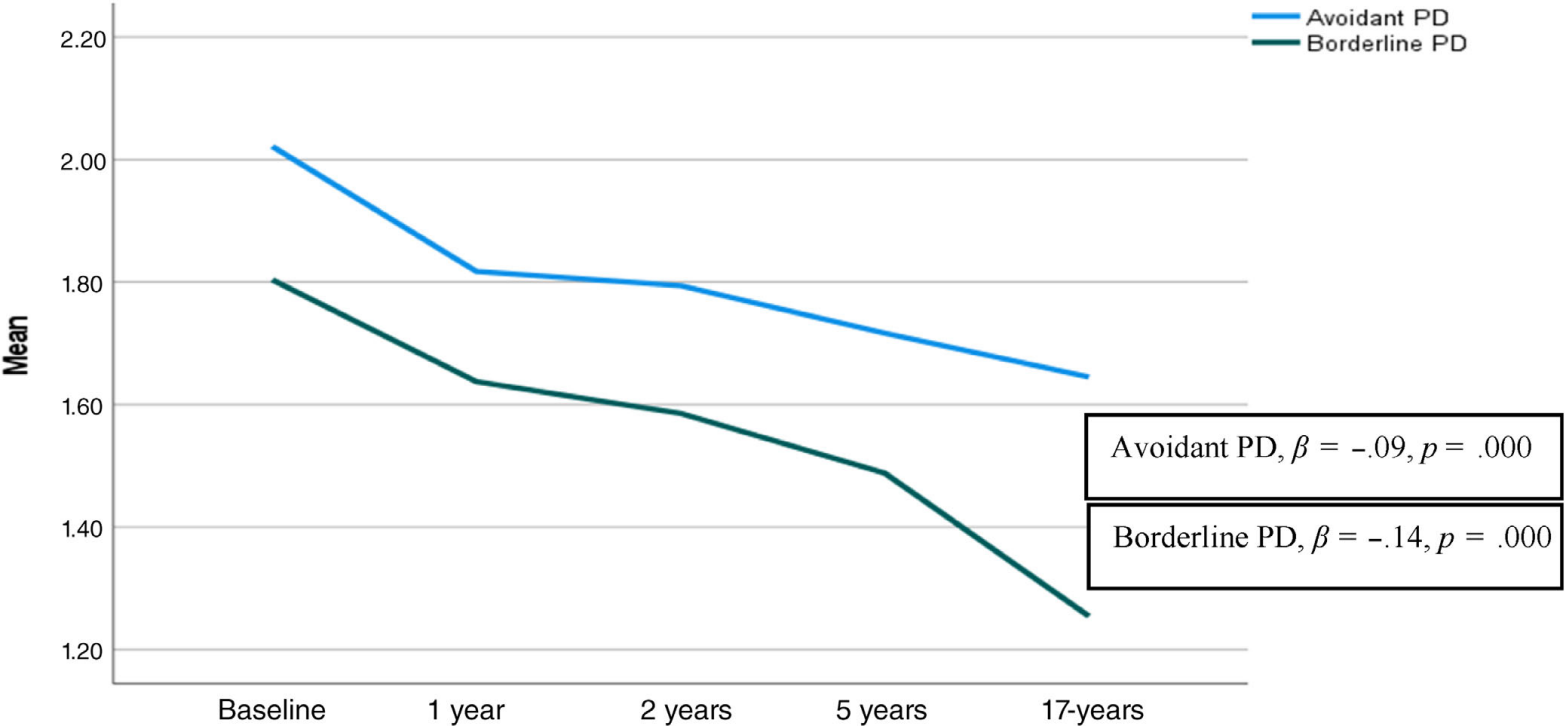
Personality indices for avoidant and borderline PD at baseline, 1‐year, 2‐year, 5‐year, and 17‐year follow‐up. Personality indices are indicated by sum scores divided by the number of items (range 1–3). PD, personality disorder

**FIGURE 2:**
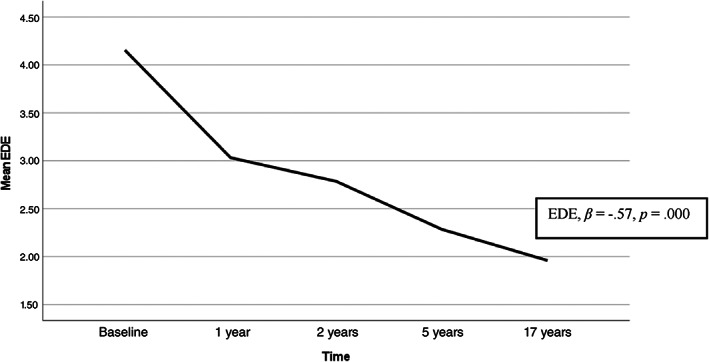
EDE scores at baseline, 1‐year, 2‐year, 5‐year, and 17‐year follow‐up (range 0–6). EDE, eating disorder examination

At baseline, 44 patients (71.0%) had one or more PD (Table 1). The number of patients with no PD increased significantly from 16 (25.8%) at baseline to 47 (75.8%) at the 17‐year follow‐up (*χ* ^2^ = 21.189, *p* < .000). The total number of PD decreased significantly from 80 at baseline to 21 at the 17‐year follow‐up (*χ* ^2^ = 90.000, *p* = .000). The most frequently observed PD were avoidant, dependent, obsessive–compulsive, paranoid, and borderline PD (Table 1). Overall, there was a steady decrease in categorical PD across all assessments (Table 1). The same pattern was evident for dimensional measures of avoidant and borderline PD (Figure 1) and global EDE scores (Figure 2). MLM analyses revealed a significant effect of time for all outcome variables (Table 2), displaying a continuous decline in both personality psychopathology and ED symptoms across 17 years of assessments. The subsample of 25 patients (40.3%) that were underweight at admission had an increase in BMI from 16.6 (SD: 1.7) to 17.3 (SD: 2.1) at discharge, at which point the baseline SCID‐II assessments were conducted. The number of patients with a BMI < 18.5 decreased to 20 (32.3%) at discharge, and among these patients, 13 (65%) had one or more PD, with a total of 29 PD diagnoses. At the 17‐year assessment only eight patients were underweight, and five (62.5%) of them fulfilled criteria for PD diagnoses, with nine diagnoses altogether.

**TABLE 1:** DSM‐IV PD during hospital treatment, at 1‐year, 2‐year, 5‐year, and 17‐year follow‐up

	Baseline *N* (%)^a^	1‐year *N* (%)^b^	2‐year *N* (%)^c^	5‐year *N* (%)^d^	17‐year *N* (%)^e^	Statistics^f^
No PD	16 (25.8)	26 (41.9)	28 (45.2)	36 (58.1)	47 (75.8)	21.189*
One PD	21 (33.9)	15 (24.2)	24 (38.7)	16 (25.8)	9 (14.5)	5.042*
Two or more PDs	23 (37.1)	19 (30.6)	7 (11.3)	9 (14.5)	6 (9.7)	11.130***
Cluster A	15 (24.2)	12 (19.4)	2 (3.2)	7 (11.3)	4 (6.5)	5.89*
Paranoid	13 (21.0)	9 (14.5)	2 (3.2)	4 (6.5)	2 (3.2)	7.69**
Schizotypal	0	1 (1.6)	0	0	0	
Schizoid	3 (4.8)	2 (3.2)	0	3 (4.8)	2 (3.2)	.000
Cluster B	14 (22.6)	12 (19.4)	11 (17.7)	4 (6.5)	0	12.07***
Histrionic	2 (3.2)	0	0	0	0	.000
Narcissistic	0	0	0	0	0	
Borderline	14 (22.6)	12 (19.4)	11 (17.7)	4 (6.5)	0	12.07**
Antisocial					0	
Cluster C	39 (62.9)	29 (46.8)	23 (37.1)	23 (37.1)	15 (24.2)	16.53**
Avoidant	28 (45.2)	22 (35.5)	20 (32.3)	18 (29.0)	9 (14.5)	14.09**
Dependent	6 (9.7)	5 (8.1)	2 (3.2)	1 (1.6)	0	4.17*
Obs/comp	14 (22.6)	11 (17.7)	5 (8.1)	8 (12.9)	8 (12.9)	1.39

Abbreviations: obs/comp, obsessive–compulsive PD; PD, personality disorders.

^a^ *N* = 60.

^b^ *N* = 61.

^c^ *N* = 59.

^d^ *N* = 61.

^e^ *N* = 62.

^f^ McNemar, first assessment compared with 17‐year assessment.

*Note*: * *p* < .05; ** *p* < .01; *** *p* < .001.

**TABLE 2:** Main effects: Fixed effects and random effects (variance–covariance) estimates for avoidant PD, borderline PD and eating disorder symptoms

Parameter	Avoidant PD	Borderline PD	EDE
Fixed effects			
Intercept	1.99 (0.08)***	1.81 (0.06)***	4.12 (0.15)***
Time	−0.09 (0.02)***	−0.14 (0.01)***	−.57 (0.06)***
Random effects			
AR1 diagonal	0.15 (0.04)***		
ARH1 rho	0.28 (0.18)	0.59 (0.07)***	0.55 (0.11)***
Unstructured	−0.02 (0.02) to 0.32 (.09)***^a^		
Variance		0.05 (0.02)–0.20 (0.04)***^b^	1.25 (0.41)–2.95 (0.79)***^c^
Intercept		0.04 (0.02)	0.20 (0.34)
Time			0.01 (0.06)
−2 log‐likelihood	408.95	199.45	1037.88

*Note*: Standard errors are in parentheses. *p < .05; **p < .01; ***p < .001.

Abbreviations: AR1, first‐order autoregressive; ARH1, heterogeneous first‐order autoregressive; EDE, eating disorder examination; PD, personality disorder.

^a^ Significance range from <.001 to nonsignificant.

^b^ Significance range from .000–to .008.

^c^ Significance range from .000– to .034.

### 3.2 ED‐PD changes on the between‐person and within‐person levels

Control variables: Neither BMI, treatment duration, illness duration, nor age at baseline were significantly associated with the outcome variables and were therefore excluded from further analyses.

Outcome variables: Based on measurement issues, only avoidant and borderline PD were included in the MLM analyses, along with EDE. There was a significant reciprocal between‐person effect, where the initial level of EDE was positively associated with the mean level of PD throughout the course, and vice versa. More specifically, baseline EDE scores displayed a significant between‐person effect on avoidant (*β* = 1.51, SE = 0.33, *t*[72.60] = 4.58, *p* < .001; Table 3) and borderline PD (*β* = 0.56, SE = 0.20, *t*[65.331] = 2.74, *p* = .008; Table 5) across the assessments. Also, initial levels of avoidant (*β* = 1.26, SE = 0.29, *t*[81.326] = 4.27, *p* < .001; Table 4) and borderline PD (*β* = 4.24, SE = 1.22, *t*[78.690] = 3.340, *p* < .001; Table 6) were positively associated with the level of EDE over the course of the follow‐up. When examining the interaction with time, we found a significant time by between‐person borderline PD effect (*β* = 1.38, SE = 0.63, *t*[235.73] = 2.20, *p* = .029; Table 6). Thus, higher initial levels of borderline PD predicted less decrease in ED symptoms over the observation period. There was no significant interaction with time for avoidant PD. No significant within‐person effects (all absolute *t* values < 0.952) in either direction were found.

**TABLE 3:** Fixed effects estimates and random effects (variance–covariance) estimates for eating disorder symptoms predicting avoidant personality disorder

Parameter	Model 1	Model 2
Fixed parameters		
Intercept	−4.49 (1.37)**	−2.94 (1.99)
Time		−0.65 (0.59)
Between‐person	1.51 (0.33)***	1.17 (0.48)*
Within‐person	−0.00 (0.03)	−0.00 (0.03)
Between‐person × time		0.14 (0.14)
Random parameters		
AR1 diagonal	0.27 (0.08)**	0.24 (0.07)***
AR1 rho	0.57 (0.14)***	0.53 (0.14)***
Intercept variance	0.07 (0.08)	0.09 (0.07)
−2 log‐likelihood	330.22	331.58

*Note*: Standard errors are in parentheses. *p < .05; **p < .01; ***p < .001.

Abbreviation: AR1, first‐order autoregressive.

**TABLE 4:** Fixed effects estimates and random effects (variance–covariance) estimates for avoidant personality disorder predicting eating disorder symptoms

Parameter	Model 1	Model 2
Fixed parameters		
Intercept	0.08 (0.60)	−0.28 (0.94)
Time		0.14 (0.33)
Between‐person	1.26 (0.29)***	1.82 (0.46)***
Within‐person	0.08 (0.23)	0.05 (0.22)
Between‐person × time		−0.24 (0.16)
Random parameters		
AR1 diagonal	2.02 (0.36)***	1.85 (0.33)***
AR1 rho	0.51 (0.08)***	0.49 (0.09)***
Time variance	0.05 (0.04)	0.05 (0.04)
−2 log‐likelihood	823.72	809.61

*Note*: Standard errors are in parentheses. ***p < .001.

Abbreviation: AR1, first‐order autoregressive.

**TABLE 5:** Fixed effects estimates and random effects (variance–covariance) estimates for eating disorder symptoms predicting borderline personality disorder

Parameter	Model 1	Model 2
Fixed parameters		
Intercept	−0.99 (0.84)	−1.32 (1.64)
Time		0.08 (0.43)
Between‐person	0.56 (0.20)**	0.76 (0.40)
Within‐person	0.02 (0.02)	0.02 (0.02)
Between‐person × time		−0.05 (0.10)
Random parameters		
Variance	0.09 (0.02)–0.28 (0.05)***	0.07 (0.01)–0.21 (0.04)***
ARH1 rho	0.63 (0.07)***	0.60 (0.06)***
−2 log‐likelihood	198.20	162.94

*Note*: Standard errors are in parentheses. ^**^ *p* < .01; ^***^ *p* < .001.

Abbreviation: ARH1, heterogeneous first‐order autoregressive.

**TABLE 6:** Fixed effects estimates and random effects (variance–covariance) estimates for borderline personality disorder predicting eating disorder symptoms

Parameter	Model 1	Model 2
Fixed parameters		
Intercept	−5.20 (2.21)*	2.21 (3.58)
Time		−2.84 (1.14)*
Between‐person	4.24 (1.22)***	0.61 (1.97)
Within‐person	−0.15 (0.26)	−0.00 (0.25)
Between‐person × time		1.38 (0.63)*
Random parameters		
AR1 diagonal	2.51 (0.29)***	2.30 (0.27)***
AR1 rho	0.60 (0.05)***	0.59 (0.05)***
−2 log‐likelihood	825.35	806.92

*Note*: Standard errors are in parentheses. *p < .05; ***p < .001.

Abbreviation: AR1, first‐order autoregressive.

## 4 DISCUSSION

A marked reduction in both personality psychopathology and ED symptoms was observed at the 17‐year follow‐up. Avoidant, borderline, dependent, paranoid, and obsessive–compulsive PD were the most frequently detected PD across assessments. Both avoidant and borderline PD at baseline were positively associated with the level of ED over the course of the follow‐up. Similarly, the initial level of ED was positively associated with the average level of these PD over time. Higher baseline levels of borderline PD were particularly disadvantageous for the long‐term outcome of ED, predicting less decline.

The PD prevalence at the end of treatment was higher than what is commonly reported for patients with ED (Friborg et al., 2014; Martinussen et al., 2017). This is particularly notable since diagnostic interviews generally yield fewer false positive cases than self‐report instruments (Cassin & von Ranson, 2005; Ramklint et al., 2010). However, PD proportions tend to be higher among inpatients as compared with outpatients or community samples. A meta‐analysis found that the ED–PD comorbidity varied from 49% in outpatients to 75% in inpatients (Rosenvinge et al., 2000). Several distinctions in our sample might be related to the elevated PD incidence, such as the average illness duration of nearly 13 years at baseline already, in addition to several unsuccessful treatment attempts. Still, no significant effect of illness duration was found when controlled for in the MLM analyses. Possibly, the severity might be better operationalized by the number of treatment attempts without sufficient effect. Several studies have concluded that symptom severity seems to play a significant role in ED–PD comorbidity (Rowe et al., 2010; Wonderlich et al., 1994), and longstanding ED is related to substantial social and psychological symptoms that might complicate the recovery process (Zipfel et al., 2000). Another factor likely to influence the PD rate is the patients' BMI, considering that low weight is associated with a higher proportion of PD (Martinussen et al., 2017). Although efforts were made to reduce the risk of overestimation of PD, a third of the sample were still underweight at the time of discharge, and this might have inflated the PD prevalence. This is a recurring methodological challenge when studying the relationship between PD and ED. Comorbid PD is associated with complications to clinical care and could possibly represent barriers to recovery that should be addressed in therapy (Simpson et al., 2021). Meanwhile, caution is required, as several of the clinical features of PD overlap with symptoms related to starvation and low weight, which could lead to misinterpretations. With this in mind, BMI was controlled for in the MLM analyses, and it did not seem to significantly impact the ED–PD comorbidity. Regardless of this, we cannot exclude the possibility that the occurrence of PD would differ had the patients achieved normal weight. The transdiagnostic nature of the sample seems to be reflected in the variety in BMI, and also in the fact that both the PD most frequently seen in restrictive AN and binge‐ED (avoidant and obsessive–compulsive PD) and the disorders which are more common among patients with binge‐eating/purging AN, BN and OSFED (borderline and paranoid PD) are present across assessments (Farstad et al., 2016).

Throughout the follow‐up period, there was a steady decline in the number of PD and the number of patients fulfilling criteria for any PD. This was particularly true for borderline PD. A 10‐year follow‐up of patients with borderline PD without concurrent ED reported similarly optimistic results and showed low rates of relapse (Gunderson et al., 2011). A contrary trend was the stability of obsessive–compulsive PD, showing no significant improvement at the 17‐year assessment. A systematic review suggests that when such PD traits are concomitant, a tailored treatment may be beneficial to reduce the risk of poor ED prognosis (Crane et al., 2007). Avoidant PD was largely reduced; however, in line with most studies on PD among patients with ED (Friborg et al., 2014; Martinussen et al., 2017), the Cluster C PD remained the most frequently diagnosed PD in the sample across all assessments. Shared personality traits between subgroups of ED and the Cluster C disorders is one way of accounting for this stability. This is supported by Klump et al. (2004), who concluded that regardless of recovery status, patients with ED display traits such as harm avoidance, lower self‐directedness, and cooperativeness. In a similar vein, a comorbid Cluster C diagnosis might represent a barrier to treatment benefits as well as attracting less clinical attention in the treatment of an ED (Jordan et al., 2014; Vrabel, Hoffart, et al., 2010). Although less prominent in our sample given the BMI criterion for hospital admission, the effects of malnutrition leading to rigidity and obsessive–compulsive behavior (Vitousek & Stumpf, 2004) are examples of how overlapping artifacts of the disorders might explain some of the PD‐ED comorbidity (von Lojewski et al., 2013).

Contrary to findings in longitudinal studies of intermediate duration (Grilo et al., 2007; Rowe et al., 2008, 2010), the patients who entered inpatient treatment with more severe avoidant or borderline PD psychopathology displayed higher average levels of ED throughout the 17‐year follow‐up, as compared with the patients with less evidence of personality disturbance. Similarly, the mean level of ED symptoms at baseline was positively associated with the mean level of the two PD across assessments.

While the between‐person effects describe a connection between mean levels of symptoms across assessments, they do not inform of how this relationship changes over time. An examination of interactions with time revealed that higher baseline levels of borderline PD predicted less ED improvement throughout the follow‐up period. This aligns with studies concluding that borderline PD seem detrimental to ED outcome (Hessler et al., 2019; Wonderlich et al., 1994), and contrasts with findings that describe similar ED recovery rates regardless of this comorbidity (Rowe et al., 2008; Zeeck et al., 2007). The poor prognosis might be explained by several factors. Many typical characteristics of borderline PD, such as suicidal ideation and behavior, might overshadow ED psychopathology of more covert nature (Cassin & von Ranson, 2005; Chen et al., 2011; Pettersen et al., 2008; Wildes et al., 2011). Also, the ED most commonly co‐occurring with borderline PD (Martinussen et al., 2017; Farstad et al., 2016; Zanarini et al., 2010), are not necessarily characterized by underweight or other noticeable signs. Consequently, maladaptive thoughts and actions might become more ingrained by the time they are detected and treated (Simpson et al., 2021). Furthermore, borderline PD is generally related to relational difficulties due to emotional dysregulation, presenting a severe obstacle in establishing the alliance essential for therapy (Hessler et al., 2019; Olofsson et al., 2020). In our case, the patients were hospitalized in a specialized ED unit, which likely gave priority to the treatment of ED rather than PD symptoms. It is possible that the present results would differ if PD had been the primary illness and objective in therapy (Zanarini et al., 2010).

The lack of statistically significant within‐person effects could be interpreted in several ways. It may indicate that there is no connection between the ED symptoms for a specific patient at one point in time and their PD level at a subsequent assessment, or vice versa. With that interpretation, the MLM results imply that the ED‐PD association simply describes related levels of symptom severity, rather than conditions making a specific impact on each other on a within‐person level over a long‐term course. Possibly, a larger sample size receiving frequent assessments at consistent time intervals might have generated significant within‐person effects. However, the number of subjects and measurements compare well with other studies that have found within‐person effects on similar variables (Johnson et al., 2017; Kelly & Tasca, 2016). One might question the assumption that a deviation from a person's typical state in terms of ED symptoms at the 5‐year assessment has an influence on their level of PD at the 17‐year follow‐up. Nonetheless, the lengthy timespan makes theoretical sense, as both disorders are characterized by protracted durations. Furthermore, the model fit was improved when applying numeric time rather than real time. This suggests that even though the time gap between measuring points is uneven, the rate of change between assessments is rather similar, meaning the symptom reduction is no more noticeable with increased amounts of time. This is consistent with previous publications showing that the majority of improvement occurred within the first 5 years after inpatient treatment, while there were few significant changes between the 5‐year and 17‐year follow‐up (Eielsen et al., 2021; Vrabel et al., 2008).

We note several weaknesses of this study. Analyses of the less common PD were not conducted due to low numbers and lack of variation in the sample. Furthermore, the relatively small sample size did not permit an examination of potentially divergent patterns of change related to the different ED diagnoses. MLM analyses might be better applied to datasets with several assessments at even intervals over a shorter time span, thereby possibly increasing the chances of detecting within‐person effects. Generalizability is limited due to the sample being Caucasian (Scandinavian) and predominately identified as female. Furthermore, on average the patients had been ill with ED for more than a decade, suggesting that the findings might not generalize to younger patients with shorter illness duration. Although the procedure aimed to reduce the risk of misdiagnoses of PD due to low BMI, the chance of overestimation of personality psychopathology among patients that are underweight cannot be fully eliminated.

This study also had a number of strengths. Conducting five repeated assessments using structured diagnostic interviews in a very long‐term follow‐up broadens the understanding of ED‐PD comorbidity. Furthermore, prospective studies of PD across decades are scarce, and using advanced analyses offers the opportunity to examine the entire dataset in a sophisticated manner. Attrition was on par with what is commonly observed in longitudinal studies of this duration.

The present findings are of clinical importance as they may indicate that ED and PD “travel together” in the sense that the severity of either disorder is positively associated with the other. This challenges the clinical heuristic that features such as impulsivity, obsessive–compulsive habits, and emotional instability should be therapeutically addressed only if they prevail after normalization of weight and nutritional status. Such a heuristic raises the risk of overlooking a PD or misinterpreting it as an artifact of the ED with the consequence that the PD could continue to sabotage the recovery process. This is particularly relevant in relation to borderline PD symptoms. The reciprocal relationship between the disorders might suggest that severity of symptoms, whether it be of PD or ED, should guide the focus in therapy rather than the diagnostic label. A thorough evaluation of both PD and ED is crucial, and a continuous focus on how they affect each other is necessary to establish meaningful treatment goals.

## AUTHOR CONTRIBUTIONS

**Hanna Punsvik Eielsen:** Investigation; project administration; writing – original draft; writing – review and editing. **KariAnne Vrabel:** Funding acquisition; supervision; writing – review and editing. **Asle Hoffart:** Formal analysis; supervision; writing – review and editing. **Øyvind Rø:** Conceptualization; methodology; writing – review and editing. **Jan H Rosenvinge:** Conceptualization; methodology; writing – review and editing.

## CONFLICT OF INTEREST

The author declares that there is no conflict of interest that could be perceived as prejudicing the impartiality of the research reported.

### ETHICS STATEMENT

This research complied with the Helsinki Declaration and was approved by the Regional Committees for Medical and Health Research Ethics (REC), Norway (REC‐number 2010/2548). Written informed consent was obtained from all patients. In agreement with the information given to the participants, we are prevented from submitting data to a public repository. In line with the ethical approval, videotapes, paper versions of present and previous interviews, in addition to the quantitative data, are all securely stored in appropriate facilities. An anonymized version of the dataset can be obtained from the corresponding author upon request and in accordance with national legislation. The study's design is registered on clinicaltrials.gov (clinical trial number NCT03968705).

## Supporting information


**Appendix S1** Supplemental File

## Data Availability

In agreement with the information given to the participants, we are prevented from submitting data to a public repository. In line with the ethical approval, videotapes, paper versions of the present and previous interviews, and the quantitative data are all securely stored in appropriate storage facilities. An anonymized version of the dataset can be obtained from the corresponding author upon request and in accordance with national legislation.
