# Supplementary figures and images for: Reciprocal relationships between personality disorders and eating disorders in a prospective 17‐year follow‐up study
Source: Int J Eat Disord. 2022 Oct 10;55(12):1753–64. doi: 10.1002/eat.23823 (PMC10092669; doi:10.1002/eat.23823)

Supplemental file. Attrition and reasons thereof at discharge, 1-, 2-, 5- and 17-year follow-up

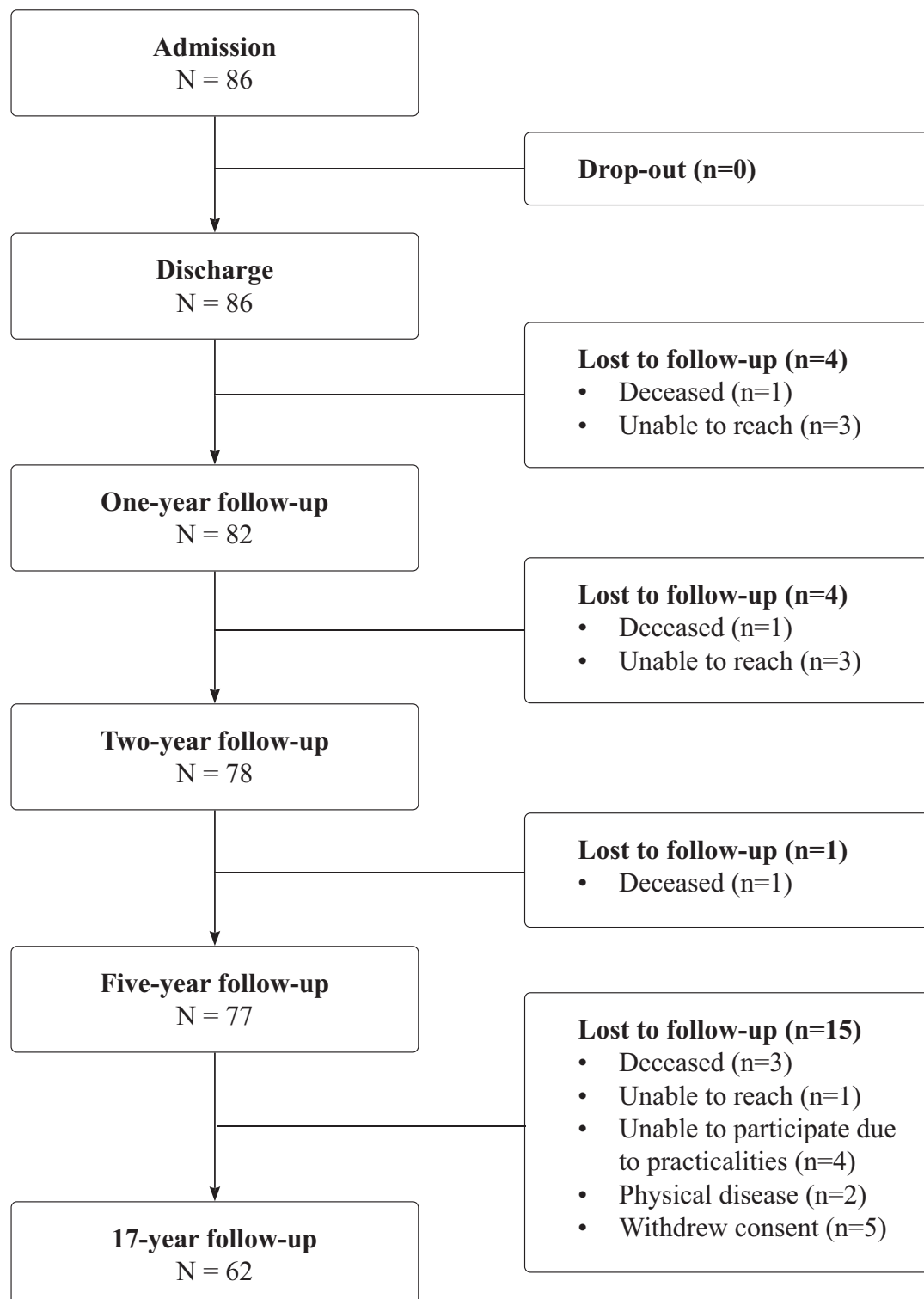

Supplement: Supplementary file 1 — Appendix S1 Supplemental File [file EAT-55-1753-s001.pdf]
